# Fiber-to-stone distance and fragment size with holmium laser lithotripsy

**DOI:** 10.1007/s00240-026-01970-x

**Published:** 2026-03-19

**Authors:** Leilane Glienke, Timothy L. Hall, Khurshid R. Ghani, William W. Roberts

**Affiliations:** 1 Department of Urology, University of Michigan, 1500 E. Medical Center Drive, Taubman Center 3879, 48109-5330 Ann Arbor, Michigan, USA; 2 Department of Biomedical Engineering, University of Michigan, Ann Arbor, MI USA

**Keywords:** Laser-fiber-to-stone distance, Stone fragment size, Laser lithotripsy, Ureteroscopy

## Abstract

Control of fragment size during laser lithotripsy has taken on increasing importance with adoption of ureteroscopic aspiration technologies. The objective of this study was to characterize the relationship between laser-fiber-to-stone distance (FSD) and the size of stone fragments produced when using Ho:YAG laser lithotripsy with fragmentation settings. Brushite crystalline aggregate model stones were treated with Ho:YAG laser with MOSES™ distance (MD) and short pulse (SP) modes. A 200 μm laser fiber was positioned at 0, 0.5, 1.0, 1.5, or 2.0 mm above the stone surface. Pulsed laser energy (0.8 J x 10 Hz) was delivered continuously while the fiber was automatically translated through a predetermined grid of points to cover the entire stone surface. Multiple passes were performed until ≥ 50% of each stone was ablated. Stone fragments were passed through sequential sieves to separate the fragments into size ranges of 2–4, 1–2, 0.5-1, and 0.25–0.5 mm. A statistically significant inverse relationship between FSD and the distribution of fragment sizes was discovered when using MD mode. For example, the risk of creating a fragment > 1 mm with FSD of 0 mm was approximately 4, 7, 27, and 27 times greater than FSD of 0.5, 1.0, 1.5, and 2.0 mm respectively. With SP mode there was no statistical difference in fragment size distribution for any FSD tested. This discovery has potential impact on laser lithotripsy techniques and provides motivation for technological developments to measure (and better manage) FSD during laser lithotripsy.

**Supplementary Information:**

The online version contains supplementary material available at 10.1007/s00240-026-01970-x.

## Introduction

Suction has recently been incorporated into ureteroscopy to enhance clearance of stone fragments and debris. Whether suction is applied through a flexible and navigable suction ureteral access sheath (FANS) or through a direct in-scope suction ureteroscope (DISS), effective use of suction requires that fragments not clog the outflow channel [1–4]. Clogging decreases efficiency of stone clearance, requires additional maneuvers to clear the channel, and presents risks from increased intrarenal pressure (IRP) [5–8]. Clogging is a complex physical phenomenon that has been rigorously studied within the realm of microfluidics and typically involves interactions between multiple fragments within a channel [9, 10]. Not surprisingly clogging was found to be dependent on the ratio of particle size to pore size [9]. Extrapolating this concept to ureteroscopy, a number of recent studies have examined the maximal size of stone fragments (or sand particles) that can successfully be aspirated through various ureteroscopic outflow channels [1–4]. Hence, it would seem to be important to control (or be able to adjust) the size of fragments produced from laser lithotripsy to reduce clogging events.

One laser lithotripsy strategy to prevent production of large stone fragments and decrease risk of clogging is to use laser types and settings designed for stone dusting as this has been shown to produce fewer larger fragments than fragmentation settings [11–13]. However, not all stones are amenable to dusting and in cases where dusting is effective, there are often residual portions of the stone not converted to dust. As the stone mass is gradually reduced, these fragments can be difficult to stabilize to complete dusting. In many scenarios, it may prove necessary (even advantageous) to use laser fragmentation rather than dusting settings. Thus, it is important to identify variables that influence the size of fragments produced from laser lithotripsy applied with fragmentation settings.

We hypothesized that laser-fiber-to-stone distance (FSD) would be inversely related to production of larger stone fragments (fragments that are problematic for aspiration). Previous research evaluating FSD has largely focused on stone ablation efficiency, typically measured as mass of stone ablated per unit time or unit energy [14–17] or crater volume measurements [17, 18]. However, little attention has been focused on the relationship between FSD and fragment size. In this study, we sought to characterize the relationship between FSD and the size of stone fragments produced when using Ho:YAG laser lithotripsy with fragmentation settings.

## Methods

**Fig. 1:**
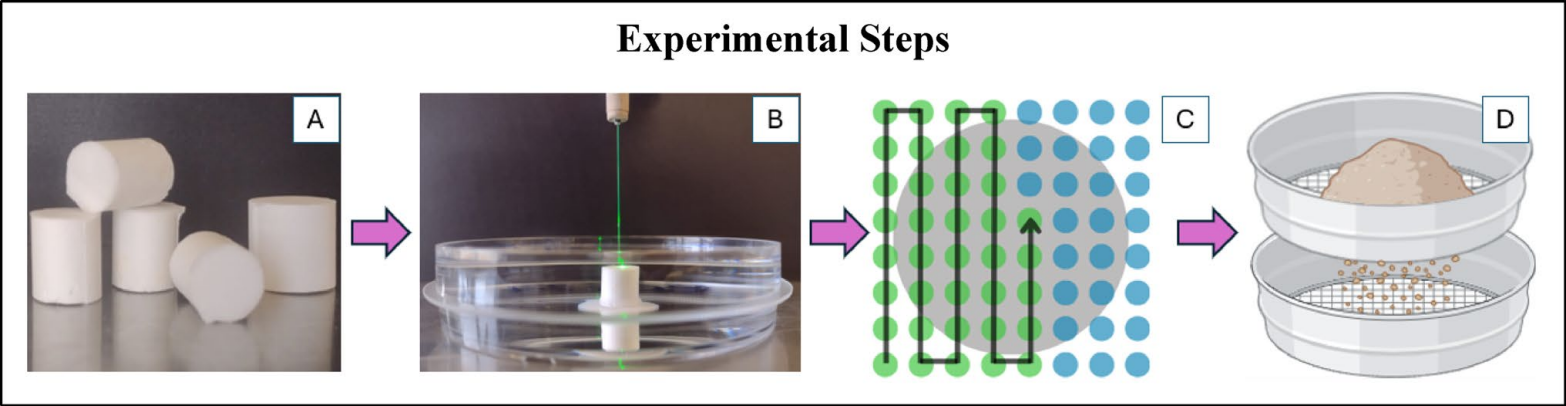
**A**) Crystalline aggregate model stones. **B**) Model stones were glued to the bottom of petri dishes and completely covered with deionized water. The laser fiber was positioned at a specified distance above the stone surface. **C**) The laser fiber was then automatically translated through a standard grid of points (1 cycle) to completely cover the surface of the stone. **D**) After a sufficient number of cycles were completed to ablate more than 50% of the stone, all stone fragments and debris were sieved to separate into size categories

Cylindrical brushite crystalline aggregate model stones (10 mm height, 7 mm diameter) [19, 20] (Fig. 1A) were weighed and fixed in a petri dish with a drop of cyanoacrylate glue. Each sample (petri dish plus glued stone) was then weighed and hydrated in deionized water for 3 h and positioned so the flat stone surface was horizontal.

A MOSES™ 200 μm D/F/L laser fiber (Boston Scientific) was positioned with its tip below the water surface at 0, 0.5, 1.0, 1.5, or 2.0 mm from the stone surface (Fig. 1B). Pulsed laser energy (0.8 J x 10 Hz) was delivered continuously from a P120H Ho:YAG laser (Boston Scientific) in either MOSES™ distance (MD) mode [full width half max (FWHM) pulse length = 342 µs] or short pulse (SP) mode [FWHM pulse length = 61 µs] (Online Resource 1). Previous research has shown the craters produced with these two modes have a greater cross-sectional area of stone ablation than other modes [17, 21] and are more likely to generate a larger number of fragments allowing more efficient testing of the hypothesis.

The laser fiber was translated across the stone surface using a 3-axis positioning system driven by an automated MATLAB program to cover an 8 × 8 grid of points (1 mm spacing between points, 0.5 s dwell time at each point, and 2.0 mm/sec velocity between points) (Fig. 1C). Before each pass through the grid the laser fiber was repositioned at the starting point and readjusted to the specified distance above the stone surface. This process was repeated until ≥ 50% of the stone mass was ablated. After every 5 passes through the grid, the fiber tip was cleaved.

At the end of each trial, stone fragments and debris were collected and passed through sequential sieves (4, 2, 1, 0.5, and 0.25 mm) to separate the fragments into size ranges of 2–4, 1–2, 0.5-1, and 0.25–0.5 mm (Fig. 1D). Fragments that would not pass through the 4 mm sieve were considered non-ablated mass. The residual/non-ablated portion of each sample was dried in a vacuum chamber at room temperature for 3 h then weighed. The smaller sized stone fragments were dried in a 37° C oven for 3 h (to prevent dispersion of the samples). The mass of ablated fragments < 0.25 mm was calculated by subtracting the mass of sieved fragments plus residual/non-ablated sample from the mass of the initial sample.

Five trials were performed at each FSD for SP and for MD mode. The percentage by mass of each range of fragment sizes was reported as mean ± standard deviation for each set of trials. The ablation rate was calculated by dividing the ablated mass of each aggregate model stone per number of cycles and reported as mean ± standard deviation. One-way ANOVA multiple comparison analyses were performed to compare between different FSD groups using Graph Pad Prism, with p value <0.05 considered to be statistically significant. In cases where the ANOVA analysis was statistically significant, pairwise comparison of groups was conducted using Tukey’s multiple comparisons test. Student’s t-test was used (Excel) to determine p-values for comparison between MD and SP mode of the mass percentage of fragments within each size range for fixed FSD.

## Results

### Trials with MD mode

**Fig. 2:**
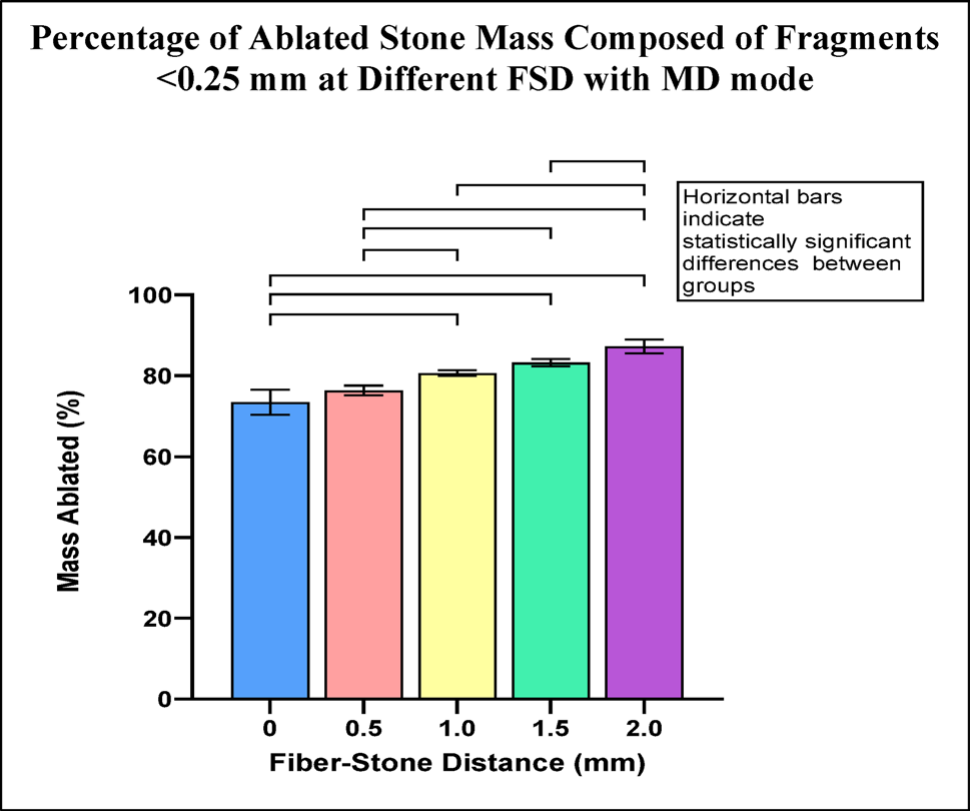
Percentage of ablated stone mass consisting of fragments <0.25 mm for each FSD after Ho:YAG laser treatment with MD mode. Error bars represent ± standard deviation. Horizontal bars indicate statistically significant differences between groups (p-values are listed in Online Resource 2)

Fragments <0.25 mm represented 73-87% of the ablated stone mass across the range of FSD with MD mode. The percentage of ablated stone mass consisting of fragments <0.25 mm increased as FSD increased. Eight of ten pairwise comparisons between FSD groups were statistically significant (Fig. 2).

**Fig. 3:**
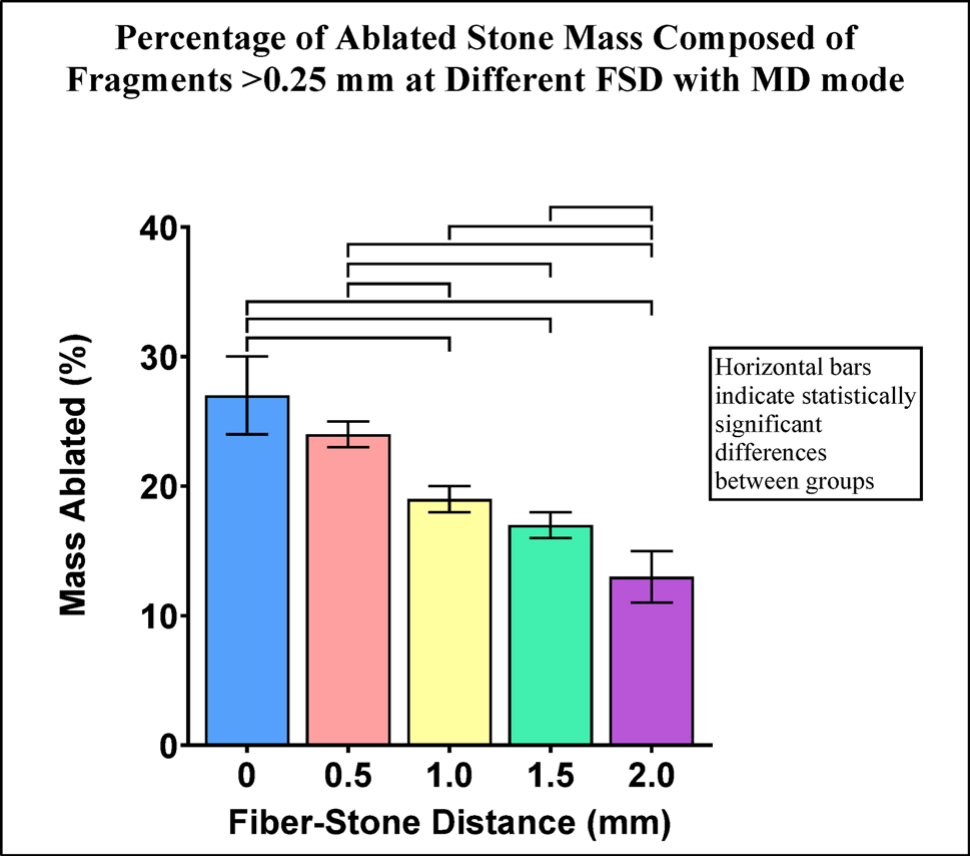
Percentage of ablated stone mass consisting of fragments >0.25 mm for each FSD after Ho:YAG laser treatment with MD mode. Error bars represent ± standard deviation. Horizontal bars indicate statistically significant differences between groups (p-values are listed in Online Resource 2)

Fragments >0.25 mm represented 13-27% of the ablated stone mass across the range of FSD with MD mode. The percentage of ablated stone mass consisting of fragments >0.25 mm decreased as FSD increased. Eight of ten pairwise comparisons between FSD groups were statistically significant (Fig. 3).

**Fig. 4:**
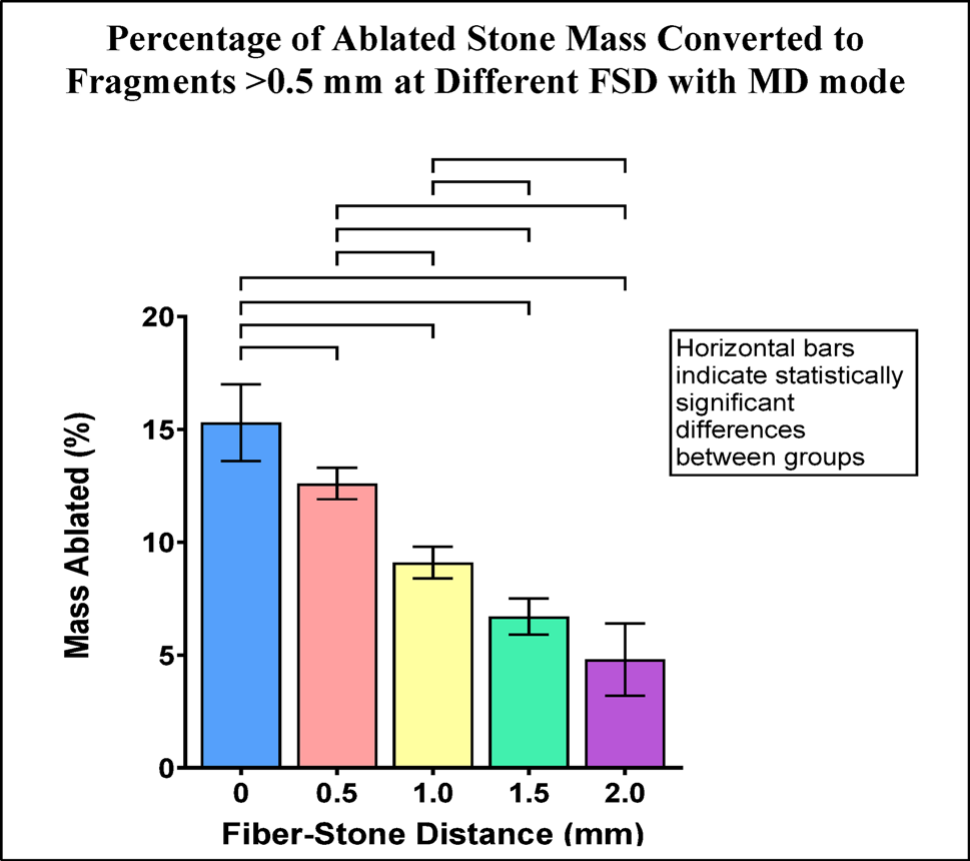
Percentage of ablated stone mass consisting of fragments >0.5 mm for each FSD after Ho:YAG laser treatment with MD mode. Error bars represent ± standard deviation. Horizontal bars indicate statistically significant differences between groups (p-values are listed in Online Resource 2)

Fragments >0.5 mm represented 5-15% of the ablated stone mass across the range of FSD with MD mode. The percentage of ablated stone mass consisting of fragments >0.5 mm decreased as FSD increased. Nine of ten pairwise comparisons between FSD groups were statistically significant (Fig. 4).

**Fig. 5:**
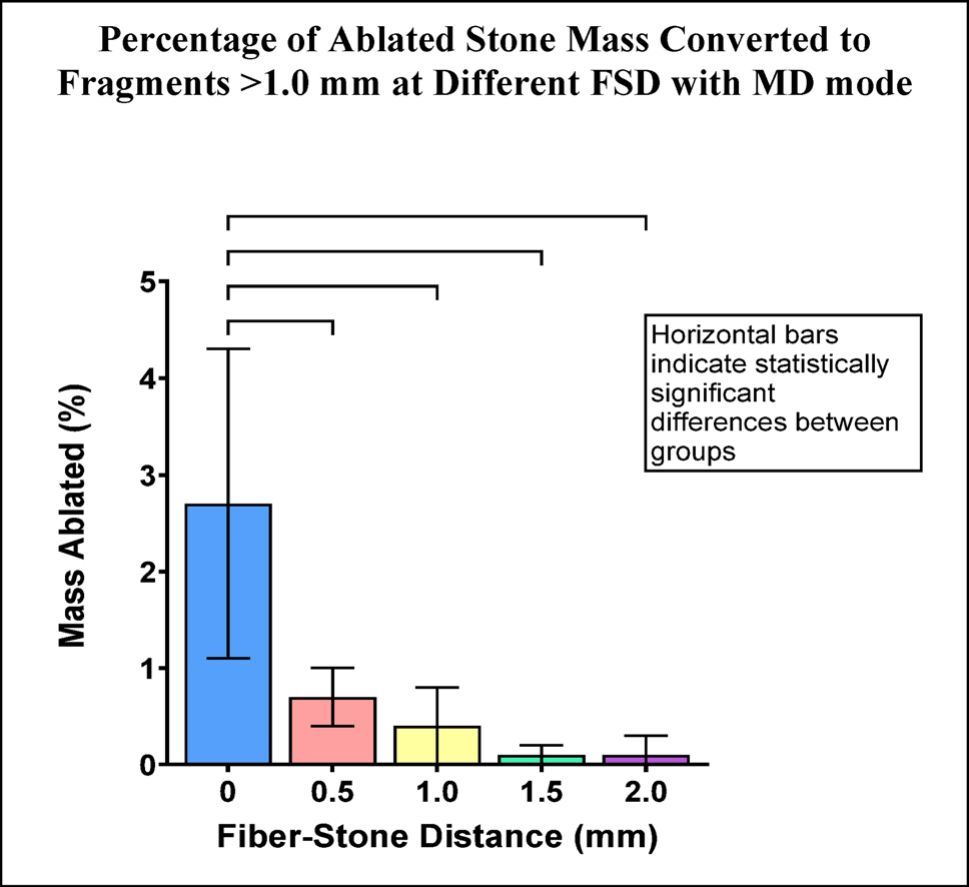
Percentage of ablated stone mass consisting of fragments >1.0 mm for each FSD after Ho:YAG laser treatment with MD mode. Error bars represent ± standard deviation. Horizontal bars indicate statistically significant differences between groups (p-values are listed in Online Resource 2)

Fragments >1.0 mm represented 3% of the ablated stone mass for FSD = 0 with MD mode. For FSD >0 the percentage of fragments >1.0 mm was <1%. Pairwise comparisons between FSD = 0 and other groups were statistically significant (Fig. 5).

**Fig. 6:**
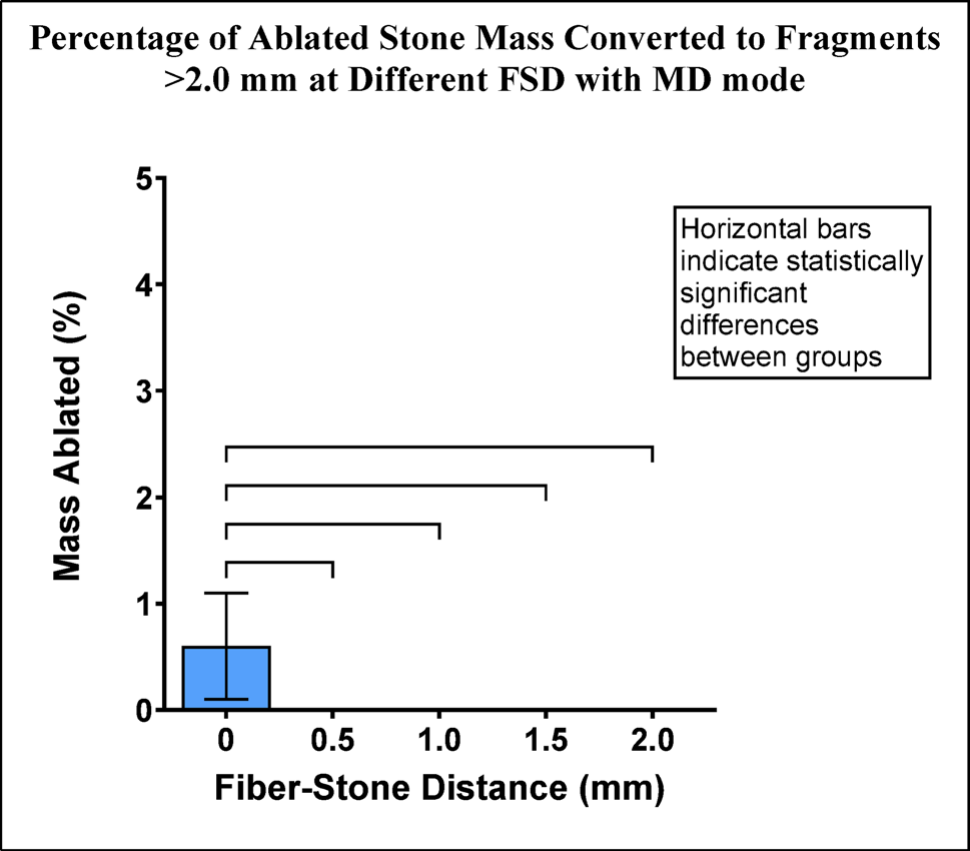
Percentage of ablated stone mass consisting of fragments >2.0 mm for each FSD after Ho:YAG laser treatment with MD mode. Error bars represent ± standard deviation. Horizontal bars indicate statistically significant differences between groups (p-values are listed in Online Resource 2)

Fragments > 2.0 mm were only produced with MD mode at FSD = 0 and represented < 1% of the mass of ablated stone. Pairwise comparisons between FSD = 0 and other groups were statistically significant (Fig. 6).

**Fig. 7:**
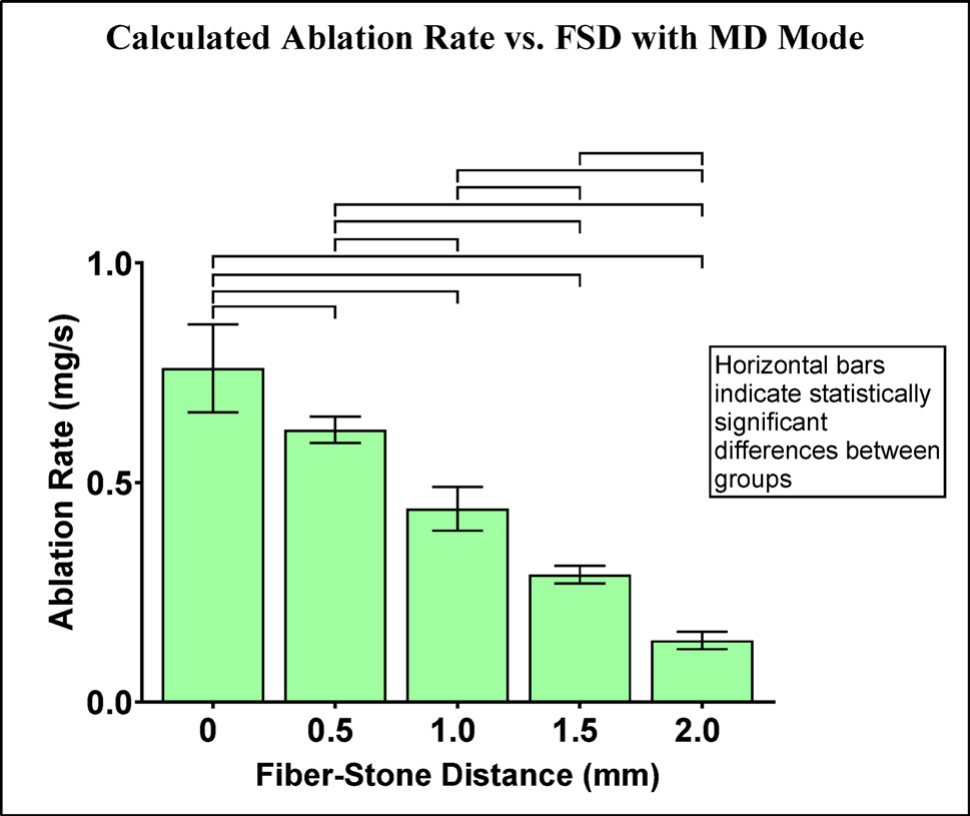
Calculated ablation rate (mg/s) with MD mode. Horizontal bars indicate statistically significant differences between groups (p-values are listed in Online Resource 4)

The calculated ablation rate with MD mode (Online Resource 3) decreased as FSD increased (Fig. 7) consistent with previous studies on ablation efficiency [15, 16]. Pairwise comparisons of ablation rate were statistically significant between all FSD groups.

### Trials with SP mode

**Fig. 8:**
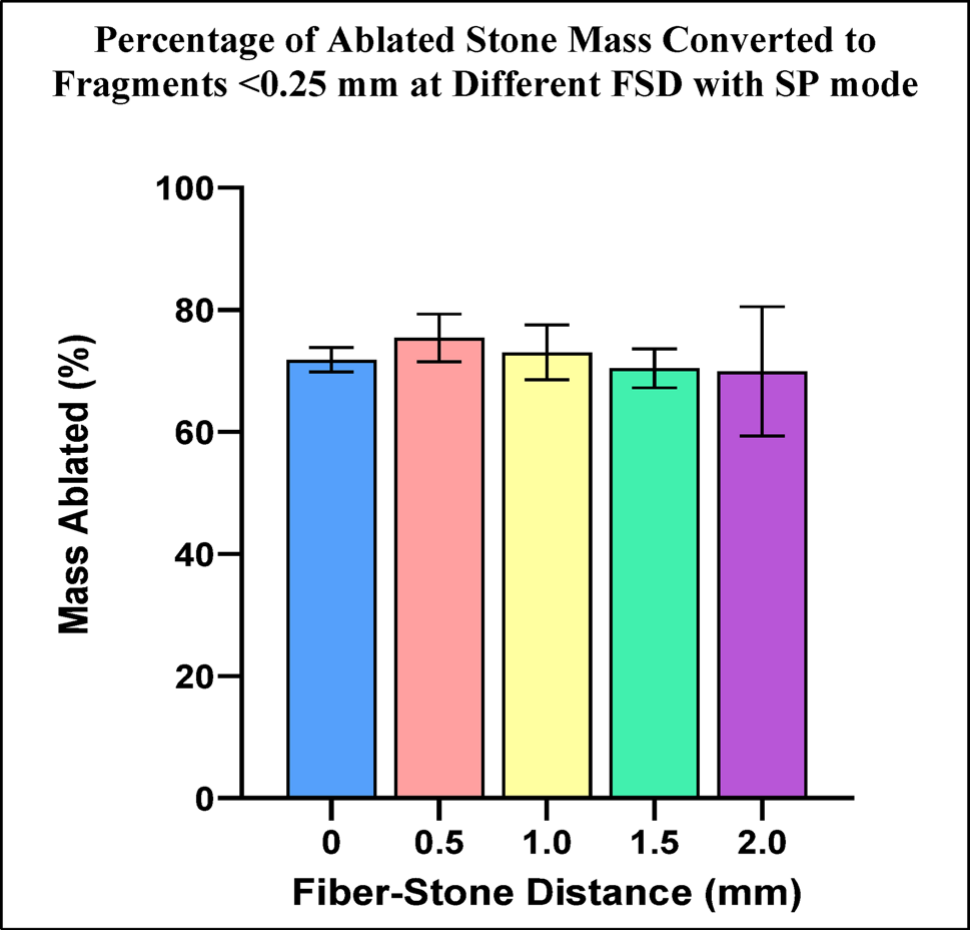
Percentage of ablated stone mass consisting of fragments <0.25 mm for each FSD after Ho:YAG laser treatment with SP mode. Error bars represent ± standard deviation. Horizontal bars indicate statistically significant differences between groups (p-values are listed in Online Resource 2)

Fragments <0.25 mm represented 70-75% of the ablated stone mass with no statistical differences based on FSD for SP mode (Fig. 8).

**Fig. 9:**
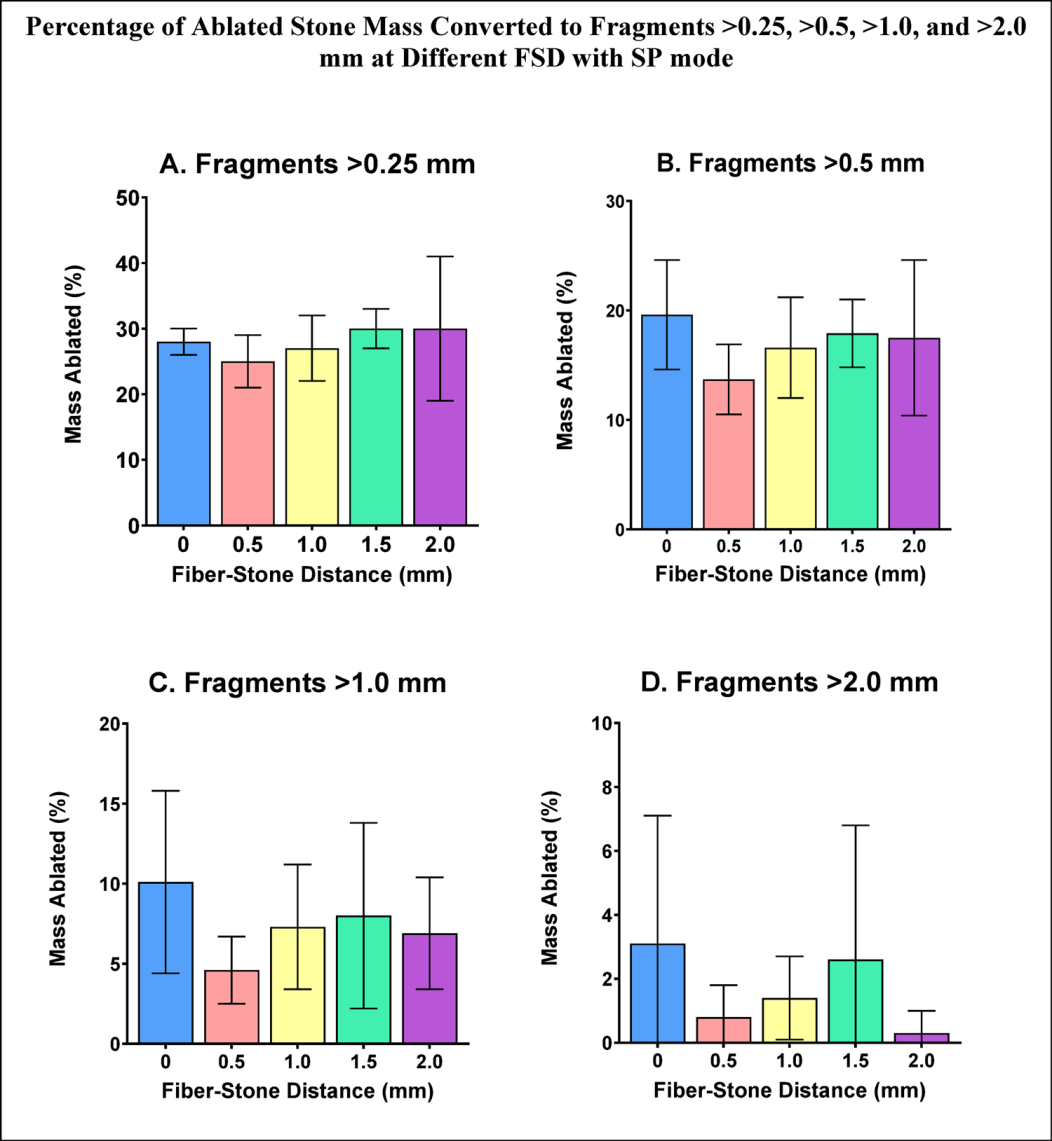
Percentage of ablated stone mass consisting of fragments >0.25 mm (A), >0.5 mm (B), >1.0 mm (C), >2.0 mm (D) at different FSD after Ho:YAG laser treatment with SP mode. Error bars represent ± standard deviation. Pairwise comparison between FSD groups within each fragment size demonstrated no statistically significant differences (Online Resource 2)

Fragments > 0.25 mm represented 25–30% of ablated stone mass with no statistical differences based on FSD for SP mode (Fig. 9A). Fragments > 0.5 mm represented 14–20% of ablated stone mass with no statistical differences based on FSD for SP mode (Fig. 9B). Stone fragments > 1.0 mm represented 5–10% of the ablated stone mass with no statistical differences based on FSD for SP mode (Fig. 9C). Stone fragments > 2.0 mm represented 0–3% of the ablated stone mass with no statistical differences based on FSD for SP mode (Fig. 9D).

**Fig. 10:**
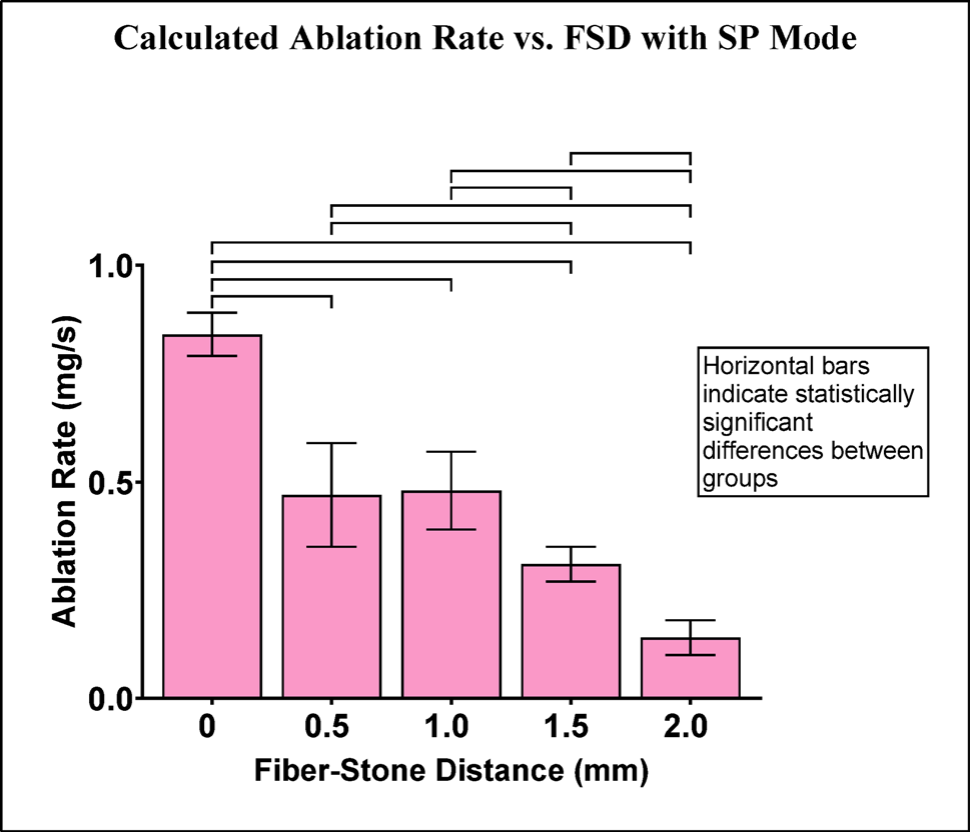
Calculated ablation rate (mg/s) with SP mode Horizontal bars indicate statistically significant differences between groups (p-values are listed in Online Resource 4)

The calculated ablation rate with SP mode (Online Resource 3) decreased as FSD increased (Fig. 10). Each pairwise comparison of FSD groups exhibited statistically significant differences in ablation rate except for comparison of 0.5 and 1.0 mm.

### Comparison of MD and SP mode

For each FSD, the mass percentage of fragments greater than 0.25, 0.5, 1, and 2 mm was less for MD compared to SP mode (Table 1). At FSD of 1, 1.5 and 2 mm the differences between MD and SP modes were statistically significant except for comparisons of fragments > 2 mm where MD mode produced no fragments (Table 1).

**Table 1:**
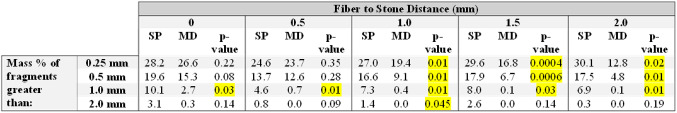
Mass % of fragments produced by short pulse and moses distance modes

## Discussion

The primary finding from this study is that increasing FSD shifts the distribution of stone fragments toward smaller sizes with Ho:YAG laser lithotripsy when employing MD mode with fragmentation settings (0.8 J x 10 Hz). Interestingly, this relationship was not seen in trials where SP mode was used and invites further exploration of laser (and pulse mode) stone interactions and the process by which stones comminute. From a clinical perspective, these results suggest that FSD is a parameter that could be utilized with MOSES™ distance mode to control the distribution of fragment sizes produced during laser lithotripsy. Discovery of this new relationship between FSD and fragment size may motivate technological developments to measure (and better manage) FSD during laser lithotripsy.

Success of stone fragment aspiration during ureteroscopy is directly related to the fragment size produced from laser lithotripsy and the size of the outflow channel. Several experimental studies have characterized fragment sizes suitable for effective aspiration with different commercially available DISS and FANS devices [1–4, 22, 23]. Fragments up to 0.25 mm were cleared with FANS:ureteroscope combinations as tight as 11/13 Fr FANS:9.5 Fr ureteroscopes [3, 4] and DISS devices with 3.6 Fr working channels [22, 23]. Fragments up to 0.5 mm were cleared with 11/13 Fr FANS:7.5 Fr ureteroscopes [3, 4] and DISS devices with a 5.1 Fr working channels [2, 22]. Fragments up to 1.0 mm were cleared with 11/13 Fr FANS:6.3 Fr ureteroscopes [3, 4]. Fragments up to 2.0 mm were cleared with 12/14 FANS:6.3 Fr ureteroscopes [4]. Thus, fragment size thresholds of 0.25, 0.5, 1.0, and 2.0 mm can all be considered relevant thresholds based on the variety of ureteroscopic equipment currently available.

The results of this current study demonstrate a statistically significant inverse relationship between FSD and the size distribution of fragments produced when using Ho:YAG laser with fragmentation settings in MD mode. As FSD increased, the distribution shifted towards smaller sized stone fragments: as FSD decreased, the distribution shifted towards larger sized stone fragments. This point is most easily understood from Fig. 5, where the risk of creating a fragment > 1 mm with FSD of 0 mm is approximately 4, 7, 27, and 27 times greater than at FSD of 0.5, 1.0, 1.5, and 2.0 mm respectively when using MD mode. Interestingly, the relationship between increasing FSD and a shift towards smaller fragment sizes with MD mode was not seen with SP mode. In fact, with SP mode there was no statistical difference in fragment size distribution for any FSD tested.

While this study is the first to our knowledge to examine the relationship between FSD and fragment size, previous publications have examined the relationship between laser pulse modes and fragment size with Ho:YAG laser lithotripsy. Tominaga et al. evaluated pop-dusting parameters (0.5 J x 80 Hz) and found MOSES™ contact (MC) mode produced a greater mass of fragments < 0.5 mm than MD or long pulse (LP) [24]. Black et al. using a fixed 2 mm FSD found that for fragmentation settings (1 J x 20 Hz), MD produced a greater percentage of stone mass < 0.25 mm and lower percentage of fragments > 2 mm compared to SP mode [25].

Data from this current study also supports a benefit to using MD mode compared to SP mode with respect to fragment size. At every FSD, a greater percentage of the ablated stone mass was in the form of fragments < 0.25 mm for MD mode compared to SP. Similarly, the percentage of ablated stone mass composed of fragments > 0.25, > 0.5, > 1.0, and > 2.0 mm was less for MD mode compared to SP mode at every FSD distance. For example, considering FSD of 1.0 mm the mass percentage of fragments > 0.25 was 19% vs. 27%, > 0.5 mm was 9% vs. 17%, > 1.0 mm was 0% vs. 7%, and > 2.0 mm was 0% vs. 1% for MD and SP mode respectively (Table 1).

One factor that may contribute to this difference between MD and SP mode is pulse length. Full-width-half-max pulse length measurements of the 0.8 J laser pulses used in this study were 342 µs for MD and 61 µs for SP (Online Resource 1), demonstrating a similar pattern to previous measurement of 1 J pulses [17, 26]. In the study by King et al., peak pressures from 1 J pulses were measured to be 62 bars for SP and 11 bar for MD mode [26]. These values can serve as approximate benchmarks for peak pressures in the current study. The higher pressure transients that occur on the surface of a stone with shorter laser pulse lengths, induce photoacoustic effects that over multiple pulses may propagate fractures in stones, which could in turn produce larger fragments [26].

The potential benefits of using larger FSD with MD mode to shift the fragment distribution toward smaller sizes, must be considered in the context of decreased ablation rate. For example, ablation rate at 2 mm FSD was 5 and 6 times lower than at 0 mm FSD for MD and SP respectively. These ablation rate findings are consistent with results from previous studies [14, 17] and are likely a consequence of greater energy absorption in the longer fluid path between the laser fiber and the stone with larger FSDs. As a result, less energy from each laser pulse reaches the stone. Lower ablation rates at larger FSDs translate into longer lasing time and greater energy use, but overall treatment time may be shorter if clogging and the associated time to clear clogs is reduced by producing smaller fragments. Optimization of treatment in the future will need to balance the speed of stone ablation against the risk of clogging and account for the size of the outflow channel being used. Effective use of FSD as a tool to control fragment size is predicated on development of effective technologies to measure FSD in real-time and perhaps even to regulate laser energy delivery within a defined FSD range. Several approaches based on reflectance and fluorescence for determination of appropriate FSD have demonstrated promising preclinical results [27, 28].

This study was focused only on Ho:YAG laser lithotripsy. We acknowledge that thulium fiber laser (TFL) and Tm:YAG lasers are also used frequently, and many authors have reported that TFL produces more dust and fewer larger fragments than Ho:YAG [12, 29]. However, in certain scenarios with TFL treatment of human stones, larger fragments can still occur [12]. It is unclear with TFL and Tm:YAG lasers if a similar relationship between FSD and fragment size might also exist. It is expected though, that stone ablation mass and ablation rate would decrease as FSD increases [30], similar to the Ho:YAG data in this study.

This current study was designed to test the scientific hypothesis that FSD is inversely related to production of larger stone fragments. While this hypothesis was confirmed and opens up a number of exciting possibilities for laser lithotripsy and aspiration of stone fragments, it is not broadly clinically applicable at this point. First, only one setting (0.8 J x 10 Hz) was tested using two pulse modes (MD, SP) in this study. While this was sufficient to demonstrate that an FSD/fragment size relationship exists for MD mode, the mechanism on which it is based and the role that different modulated and unmodulated pulse modes play, needs further exploration. Second, brushite crystalline aggregate model stones rather than human stones were used in this study. This was partially necessitated by the need to have uniform stone samples and flat stone surfaces across all experimental trials. This stone model exhibits a comminution behavior more similar to human stones than BegoStone [19] but even so, studies to validate the FSD/fragment size relationship are needed with human stones. Third, the model stones were fixed to a petri dish to prevent movement during ablation. This approach allows for consistent delivery of laser energy and precise control of FSD during trials needed to test the proposed hypothesis. This degree of stone fixation though, is not typical clinically and applicability of the findings to mobile stones will also need to be explored.

## Conclusion

Control of fragment size during laser lithotripsy has taken on increasing importance with the advent of ureteroscopic aspiration technologies. Data from this study demonstrates that increasing FSD resulted in a greater percentage of ablated stone mass in smaller fragment size categories when using Ho:YAG laser lithotripsy with MD mode and fragmentation settings (0.8 J x 10 Hz). This discovery has potential impact on laser lithotripsy techniques and provides motivation for technological developments to measure (and better manage) FSD during laser lithotripsy.

## Electronic Supplementary Material

Below is the link to the electronic supplementary material.

Supplementary Material 1

## Data Availability

Data is available by contacting the Senior Author.
