# Supplementary material for: Fiber-to-stone distance and fragment size with holmium laser lithotripsy
Source: Urolithiasis. 2026 Mar 19;54(1):69. doi: 10.1007/s00240-026-01970-x (PMC13002736; doi:10.1007/s00240-026-01970-x)
Supplement: Supplementary file 1 — Supplementary Material 1 [file 240_2026_1970_MOESM1_ESM.docx]

**Supplementary Information:**

**Fiber-to-Stone Distance and Fragment Size**

**with Holmium Laser Lithotripsy**

Leilane Glienke^1^, Timothy L Hall^2^, Khurshid R Ghani^1^, William W Roberts^1^

Departments of Urology^1^ and Biomedical Engineering^2^

University of Michigan, Ann Arbor, MI, USA

**Correspondence:**

William W. Roberts, MD

Department of Urology, University of Michigan

1500 E. Medical Center Drive, Taubman Center 3879

Ann Arbor, Michigan 48109-5330

E-mail: willrobe@med.umich.edu

**Supplementary Tables**

**Online Resource 1:**

**Online Resource 1.** Normalized temporal profiles of 0.8 J pulses (SP and MD) from a P120H Ho:YAG laser delivered through a 200 µm D/F/L laser fiber in air were generated using a photodetector (DET05D2, Thorlabs) and Picoscope 4224 (PicoTechnology). Tracings represent the average of 64 consecutive pulses fired at 10 Hz.

**Online Resource 2:**

**P-values from pairwise comparisons of mass percentage of fragments of specified size for different FSD in trials of MD or SP mode.**

|  | **Fragment Size** | | | | | | | | | |
| --- | --- | --- | --- | --- | --- | --- | --- | --- | --- | --- |
|  | **<0.25 mm** | | **>0.25 mm** | | **>0.5 mm** | | **>1.0 mm** | | **>2.0 mm** | |
|  | MD | SP | MD | SP | MD | SP | MD | SP | MD | SP |
| Comparison of FSD (mm) |  |  |  |  |  |  |  |  |  |  |
| 0 to 0.5 | NS | NS | NS | NS | 0.01 | NS | 0.004 | NS | 0.003 | NS |
| 0 to 1.0 | <0.0001 | NS | <0.0001 | NS | <0.0001 | NS | 0.0009 | NS | 0.003 | NS |
| 0 to 1.5 | <0.0001 | NS | <0.0001 | NS | <0.0001 | NS | 0.0002 | NS | 0.003 | NS |
| 0 to 2.0 | <0.0001 | NS | <0.0001 | NS | <0.0001 | NS | 0.0002 | NS | 0.003 | NS |
| 0.5 to 1.0 | 0.007 | NS | 0.003 | NS | 0.001 | NS | NS | NS | NS | NS |
| 0.5 to 1.5 | <0.0001 | NS | <0.0001 | NS | <0.0001 | NS | NS | NS | NS | NS |
| 0.5 to 2.0 | <0.0001 | NS | <0.0001 | NS | <0.0001 | NS | NS | NS | NS | NS |
| 1.0 to 1.5 | NS | NS | NS | NS | 0.03 | NS | NS | NS | NS | NS |
| 1.0 to 2.0 | <0.0001 | NS | 0.0003 | NS | 0.0001 | NS | NS | NS | NS | NS |
| 1.5 to 2.0 | 0.01 | NS | 0.02 | NS | NS | NS | NS | NS | NS | NS |

**Online Resource 2:** P-values associated with the pairwise comparisons of FSD groups in Figures 2-6(MD mode) and Figure 8 and 9 (SP mode).

**Online Resource 3:**

**Calculated Ablation Rate for 0.8Jx10Hz**

| Moses Distance | | | | | | | | |
| --- | --- | --- | --- | --- | --- | --- | --- | --- |
| FSD  (mm) | Mean ± SD of Mass Ablated  (mg) | | | Mean ± SD of Energy Applied  (KJ) | | Mean ± SD of Laser on Time  (s) | | Calculated Ablation Rate (mg/s) |
| 0 | 400 ± 30 | | | 4.3 ± 0.4 | | 533 ± 51 | | 0.76 ± 0.10 |
| 0.5 | 410 ± 20 | | | 5.3 ± 0.2 | | 660 ± 31 | | 0.62 ± 0.03 |
| 1.0 | 390 ± 20 | | | 7.1 ± 0.6 | | 889 ± 70 | | 0.44 ± 0.05 |
| 1.5 | 370 ± 10 | | | 10.1 ± 0.6 | | 1257 ± 74 | | 0.29 ± 0.02 |
| 2.0 | 260 ± 30 | | | 15.0 ± 0.2 | | 1880 ± 31 | | 0.14 ± 0.02 |
| Short Pulse | | | | | | | | |
| FSD  (mm) | | Mean ± SD of Mass Ablated (mg) | Mean ± SD of Energy  (KJ) | | Mean ± SD of Laser on Time  (s) | | Calculated Ablation Rate (mg/s) | |
| 0 | | 430 ± 20 | 4.2 ± 0.4 | | 521 ± 48 | | 0.84 ± 0.05 | |
| 0.5 | | 410 ± 20 | 7.5 ± 1.8 | | 940 ± 225 | | 0.47 ± 0.12 | |
| 1.0 | | 400 ± 20 | 6.9 ± 1.7 | | 864 ± 207 | | 0.48 ± 0.09 | |
| 1.5 | | 370 ± 20 | 9.4 ± 0.9 | | 1181 ± 118 | | 0.31 ± 0.04 | |
| 2.0 | | 300 ± 60 | 17.3 ± 1.0 | | 2159 ± 127 | | 0.14 ± 0.04 | |

**Online Resource 3.** Calculated Ablation rate (mg/s) for FSD groups with Ho:YAG 08J x 10Hz Moses Distance and Short Pulse modes.

**Online Resource 4:**

**P-values from pairwise comparisons of calculated ablation rate**

**for different FSD in trials of MD or SP mode.**

|  | **MD** | **SP** |
| --- | --- | --- |
| Comparison of FSD (mm) |  |  |
| 0 to 0.5 | 0.004 | <0.0001 |
| 0 to 1.0 | <0.0001 | <0.0001 |
| 0 to 1.5 | <0.0001 | <0.0001 |
| 0 to 2.0 | <0.0001 | <0.0001 |
| 0.5 to 1.0 | 0.0003 | NS |
| 0.5 to 1.5 | <0.0001 | 0.02 |
| 0.5 to 2.0 | <0.0001 | <0.0001 |
| 1.0 to 1.5 | 0.002 | 0.01 |
| 1.0 to 2.0 | <0.0001 | <0.0001 |
| 1.5 to 2.0 | 0.002 | 0.01 |

**Online Resource 4:** P-values associated with the pairwise comparisons of ablation rate (mg/s) for FSD groups in Figures 7 and 10 (MD and SP mode).
